# Patterns in topical treatment use for eczema flare-ups in children: a secondary analysis of the Best Emollients for Eczema (BEE) trial

**DOI:** 10.1093/skinhd/vzaf112

**Published:** 2026-03-17

**Authors:** Katherine Memory, Stephanie MacNeill, Kim Thomas, Miriam Santer, Matthew Ridd

**Affiliations:** Population Health Sciences Institute, Bristol Medical School, University of Bristol, Bristol, UK; Bristol Trials Centre, Bristol Medical School, University of Bristol, Bristol, UK; Centre of Evidence Based Dermatology, School of Medicine, University of Nottingham, Nottingham, UK; Primary Care & Population Sciences, Faculty of Medicine, University of Southampton, Southampton, UK; Population Health Sciences Institute, Bristol Medical School, University of Bristol, Bristol, UK

## Abstract

This study sought to describe use of topical treatments during eczema maintenance and flare-ups, using data from the Best Emollients for Eczema (BEE) trial. Findings showed that flare-ups were common, emollient use was generally high and that whilst topical corticosteroid use increased marginally during flare-ups, their overall use remained low. Our work is a reminder that parents may under-treat their child’s eczema flare-ups with topical corticosteroids, and that clinicians should always ask and advise about flare-up management, addressing any barriers to treatment use.

Dear Editor, Eczema (atopic dermatitis) is a chronic relapsing–remitting condition. Guidelines recommend daily emollient use to prevent flare-ups and topical corticosteroids (TCS) as required to treat flare-ups.^1^ However, little is known about how often these topical therapies are applied during clinical trials.

Using data from the Best Emollients for Eczema (BEE) trial^2^ of children aged <12 years old, we sought to describe the number of flare-ups and explore differences in topical therapy use before and after them. Participants in BEE were randomly allocated to lotion, cream, gel or ointment type of emollient, with written directions to apply ‘twice daily and when required’, along with a one-page information leaflet with a link to a short video on how to apply emollients. All other treatments were unchanged, including TCS being prescribed by their usual healthcare provider as clinically indicated. Parents completed weekly questionnaires for 16 weeks about eczema severity and topical therapy use. Eczema severity was measured using the Patient-Orientated Eczema Measure (POEM, total score 0–28: 0–2 indicates clear, 3–7 mild, 8–16 moderate, 17–28 severe).^3^ With no agreed definition of eczema flare-ups, we chose a POEM increase ≥3 from the previous week.^4^ A flare-up was defined as worsening eczema symptoms, irrespective of the previous week’s eczema severity, for example POEM ≥3 for two consecutive weeks was considered as two flares rather than worsening of a pre-existing flare-up.

Using Stata version 17.0 (StataCorp, Cary, NC, USA), descriptive statistics outlined flare-ups and topical treatment use. Differences in included and excluded participants were confirmed by a χ^2^ test for categorical data, a Mann–Whitney *U* test for nonparametric data, and a *t*-test for parametric data. A Kruskal–Wallis test explored differences in mean number of flare-ups between different baseline eczema severities. Student *t*-tests explored mean differences in topical therapy use in the week before and after the first and any flare-ups.

Of the original 550 participants, we excluded 100 because 23 did not return their weekly questionnaires at any time-point and 77 did not provide any TCS or emollient use and/or did not provide any paired POEM scores (to allow for flare-ups calculation). We found no significant differences in baseline characteristics (sex, age, ethnicity, socioeconomic background, baseline eczema severity or treatment arm) between excluded and included participants.^5^ Of the 450 included participants, 213 (47.3%) were of female sex and 392 (87.1%) were White; median age was 4 [interquartile range (IQR) 2–8] and the mean (SD) baseline POEM score was 9.2 (5.5).

Overall, 88.4% (*n* = 6759/7650, including baseline measure) of weekly POEMs were completed. Of these, there were 5972 pairs [median 15 (IQR 10–16) pairs per participant] of consecutive completed weekly POEMs. A total of 63.5% (*n* = 4571/7200) of weekly emollient use questionnaires and 86.3% (*n* = 6212/7200) of weekly TCS use questionnaires were completed, with 38.1% (*n* = 2369/6212) reporting at least 1 day of TCS use during the trial.

While 18.7% (*n* = 84/450) did not report any flare-ups, 81.3% (*n* = 366/450) of participants reported a total of 960 flare-ups (Table 1). Flare-up frequency ranged from 1 (*n* = 94/450; 20.9%) to 7 (*n* = 4/450; 0.9%) (Figure 1). The median number of flare-ups was 2 (IQR 1–3), with a similar distribution of total number of flare-ups across different baseline severities [H(3) = 0.97, *P* = 0.81, adjusted for ties] (Figure 2). Across weeks 1–16, there was a similar distribution of reported flare-ups each week ranging from 33 [week 14 *n* = 33/295 completed consecutive POEMS; 11.2%)] to 93 [week 1 (*n* = 93/432 completed consecutive POEMs; 21.5%)] (Figure 3). By treatment allocation, the total numbers of flare-ups were lotion (*n* = 231/960; 24.1%), cream (*n* = 251/960; 26.1%), gel (*n* = 260/960; 27.1%) and ointment (*n* = 218/960; 22.7%).

**Figure 1:**
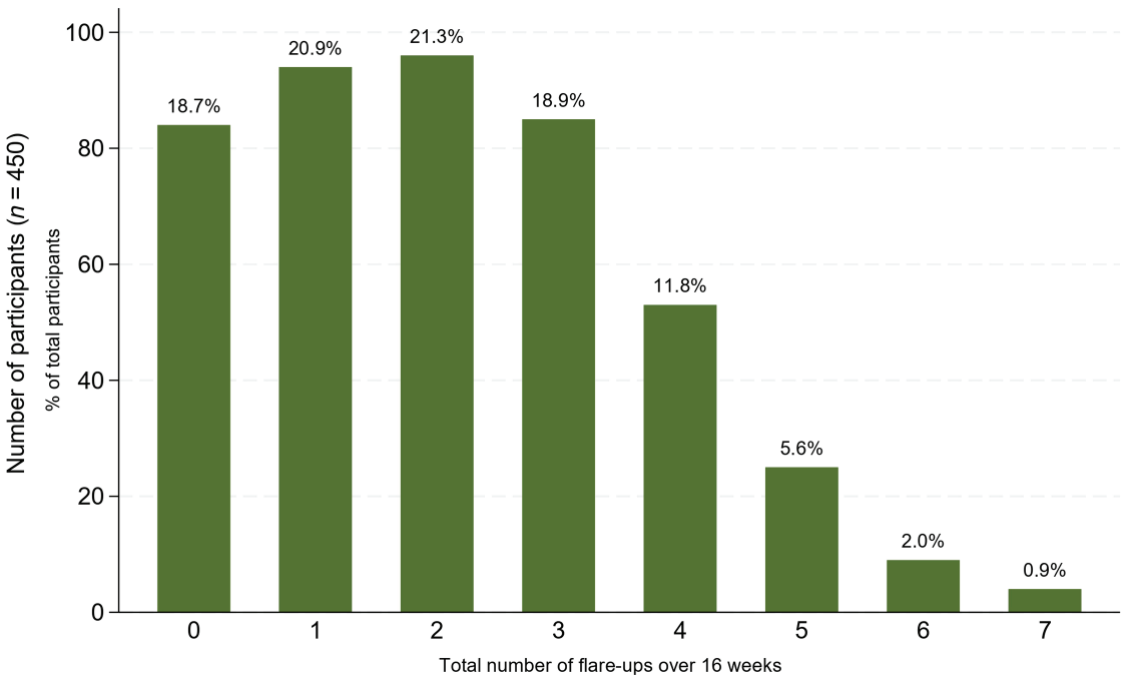
Distribution of flare-ups.

**Figure 2:**
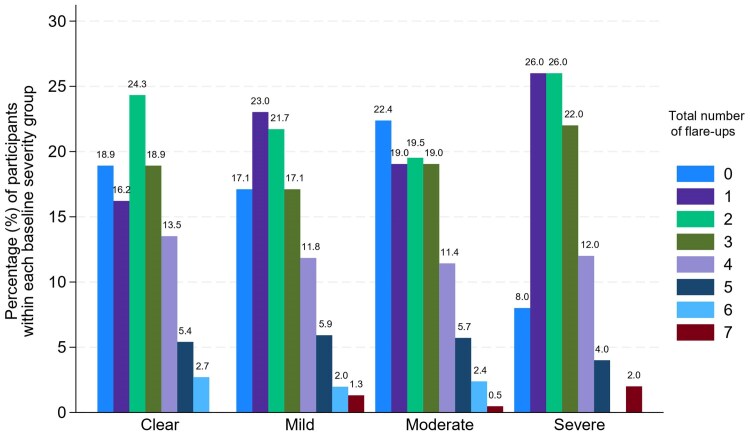
Total number of flare-ups reported by each baseline eczema severity group, as a percentage of individuals within each group.

**Figure 3:**
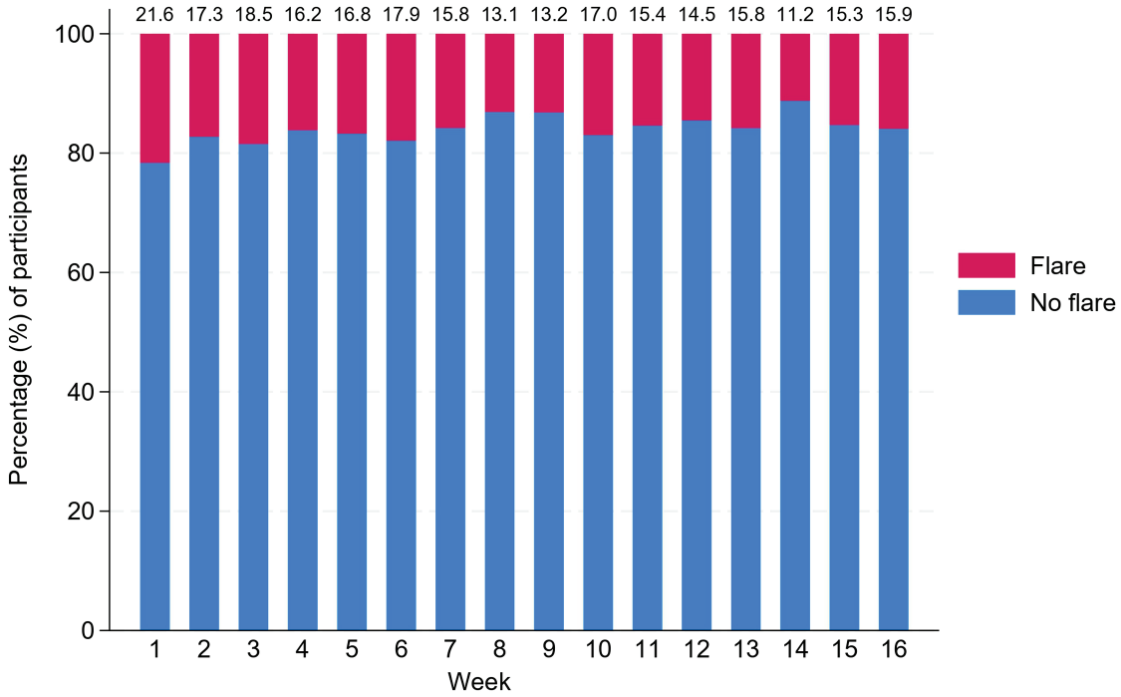
Percentage of participants reporting a flare by each week throughout the trial.

**Table 1:** Flare-ups by categorized baseline eczema severity

	Baseline eczema severity					
Number of flare-ups	Clear (*n* = 37)	Mild (*n* = 152)	Moderate (*n* = 210)	Severe (*n* = 50)	Missing (*n* = 1)	Total (*n* = 450)
0	7 (18.9)	26 (17.1)	47 (22.4)	4 (8.0)	0 (0.0)	84 (18.7)
1	6 (16.2)	35 (23.0)	40 (19.0)	13 (26.0)	0 (0.0)	94 (20.9)
2	9 (24.3)	33 (21.7)	41 (19.5)	13 (26.0)	0 (0.0)	96 (21.3)
3	7 (18.9)	26 (17.1)	40 (19.0)	11 (22.0)	1 (100)	85 (18.9)
4	5 (13.5)	18 (11.8)	24 (11.4)	6 (12.0)	0 (0.0)	53 (11.8)
5	2 (5.4)	9 (5.9)	12 (5.7)	2 (4.0)	0 (0.0)	25 (5.6)
6	1 (2.7)	3 (2.0)	5 (2.4)	0 (0.0)	0 (0.0)	9 (2.0)
7	0 (0.0)	2 (1.3)	1 (0.5)	1 (2.0)	0 (0.0)	4 (0.9)
Total flare-ups	81 (8.4)	328 (34.2)	453 (47.2)	113 (11.8)	1 (0.1)	960 (100)
Number of flare-ups	2 (1–3)	2 (1–3)	2 (1–3)	2 (1–3)	3 (3–3)	2 (1–3)

Total number of flare-ups reported by participants during trial, and flare-up count grouped by baseline eczema severity. Data are presented as *n* (%) or median (IQR). Flare defined as POEM score increase ≥3 from previous week. IQR, interquartile range; POEM, patient-orientated eczema measure, categorized as: clear, 0–2; mild, 3–7; moderate, 8–16; severe, 17–28.

The total median number of days of any emollient use was 7 (IQR 4–7) across all weeks and 7 (IQR 5–7) during flare-up weeks. There was no difference in the mean (SD) number of days of emollient use in the week before and the week after participants’ first flare-ups [0.26 (1.86); *P* = 0.08] or after any flare-up [0.09 (1.72); *P* = 0.24] (Table 2). Total median number of days of TCS use was 0 (IQR 0–2) days across all weeks and 0 (IQR 0–3) during flare-up weeks. Participants used a mean (SD) 0.43 (1.82) (*P* < 0.001) and 0.39 (1.91) (*P* < 0.001) more days per week of TCS in the week after their first flare-up and any flare-up, respectively, when compared with the week before.

**Table 2:** Differences in mean emollient and topical corticosteroid (TCS) use before and after the first and any flare-up

Flare-up definition	Topical treatment	*n* ^a^	Mean (SD) days of use during week before flare-up	Mean (SD) days of use during week after flare-up	Mean difference (SD)	Confidence interval	*P*-value
First flare-up	Emollient use	161	5.70 (1.85)	5.96 (1.76)	0.26 (1.86)	−0.03, 0.55	0.08
(first reported POEM ≥3 previous week)	TCS use	249	1.06 (1.85)	1.49 (2.04)	0.43 (1.82)	0.20, 0.65	<0.001
Any flare-up	Emollient use	501	5.70 (1.99)	5.79 (1.91)	0.09 (1.72)	−0.06, 0.24	0.24
(POEM ≥3 previous week)	TCS use	750	1.30 (1.99)	1.69 (2.22)	0.39 (1.91)	0.25, 0.54	<0.001
Any flare-up	Emollient use	446	5.65 (1.99)	5.79 (2.03)	0.17 (1.67)	0.01, 0.33	0.03
(POEM ≥3 preceded by nonflare week)	TCS use	666	1.24 (1.95)	1.65 (2.21)	0.41 (1.87)	0.27, 0.55	<0.001

Parents reported on the number of days they used their allocated emollient type, other nonallocated emollient types, and/or TCS over the last week. POEM, Patient-Orientated Eczema Measure, reports on frequency of seven eczema symptoms on a 5-point scale in the previous week (total score 0–28; higher score denotes worse disease). ^a^Number of individuals who reported a flare-up and provided topical treatment use for the week before/after flare-up. Student’s *t*-test used for calculation of mean differences.

When considering differences in use between allocated treatment arms, Anova testing showed no difference in mean emollient use before/after first flare-up [F(3157) = 1.11, *P* = 0.35] or any flare-up [F(3497) = 0.98, *P* = 0.40]. There was also no difference between groups for TCS use with first flare-up [F(3245) = 0.76, *P* = 0.52] or any flare-up [F(3746) = 0.51, *P* = 0.67].

To assess how changing the definition of flare-up altered our results, we repeated the analysis and excluded a ‘flare-up’ if the POEM change was ≥3 in two consecutive weeks. Total flares decreased from 960 to 865. There was no change in emollient or TCS use (Table 2).

In summary, we found that flare-ups were common, with a median of two flare-ups per individual over 16 weeks, with a similar distribution of flare-ups by baseline eczema severity. Mean number of days of TCS but not emollient use increased marginally following a flare-up.

The absence of an increase in emollient use during flare-ups may be either because emollient use was already high or because of a limitation in how emollient use was captured (any use each day rather than number of times applied or quantity used).

Data on real-world use of TCS is limited and our results suggest that parents applied TCS for only 2 days during flare-ups, lower than the 7–14 days as recommended.^1^ This could be because symptom relief is adequate after short treatment bursts, or because parents are under-treating due to concerns about TCS side effects. We are unable to comment on effectiveness in relation to TCS potency, the specific days on which TCS were applied or the number of times applied per day, because the questionnaire did not ask for this level of detail. Therefore, we cannot comment on the duration of TCS use before or after flare-ups.

Further details on topical treatment use are outlined in our paired paper.^5^ Findings from both studies are limited by participants being unmasked to treatments and the reliance on self-report questionnaires which may have elicited socially desirable responses, that is, participants were told to apply emollients at least twice daily. However, details on the frequency and quantity of topical treatment use, including objective assessment of eczema severity, were not possible to obtain in this pragmatic trial.

Our work is a reminder to clinicians that parents may under use TCS in the treatment of their children’s eczema, and to always ask and advise about flare-up treatment, addressing any barriers to the appropriate use of TCS.

## Data Availability

The authors will support any reasonable request for the data that underpins the findings of this study in line with the original BEE trial data sharing agreement.
